# Proactive Fault Prediction of Fog Devices Using LSTM-CRP Conceptual Framework for IoT Applications

**DOI:** 10.3390/s23062913

**Published:** 2023-03-08

**Authors:** Sabireen H, Neelanarayanan Venkataraman

**Affiliations:** School of Computer Science and Engineering, Vellore Institute of Technology, Chennai 600127, India

**Keywords:** fog environment, failure prediction, reliability, deep learning network

## Abstract

Technology plays a significant role in our daily lives as real-time applications and services such as video surveillance systems and the Internet of Things (IoT) are rapidly developing. With the introduction of fog computing, a large amount of processing has been done by fog devices for IoT applications. However, a fog device’s reliability may be affected by insufficient resources at fog nodes, which may fail to process the IoT applications. There are obvious maintenance challenges associated with many read-write operations and hazardous edge environments. To increase reliability, scalable fault-predictive proactive methods are needed that predict the failure of inadequate resources of fog devices. In this paper, a Recurrent Neural Network (RNN)-based method to predict proactive faults in the event of insufficient resources in fog devices based on a conceptual Long Short-Term Memory (LSTM) and novel Computation Memory and Power (CRP) rule-based network policy is proposed. To identify the precise cause of failure due to inadequate resources, the proposed CRP is built upon the LSTM network. As part of the conceptual framework proposed, fault detectors and fault monitors prevent the outage of fog nodes while providing services to IoT applications. The results show that the LSTM along with the CRP network policy method achieves a prediction accuracy of 95.16% on the training data and a 98.69% accuracy on the testing data, which significantly outperforms the performance of existing machine learning and deep learning techniques. Furthermore, the presented method predicts proactive faults with a normalized root mean square error of 0.017, providing an accurate prediction of fog node failure. The proposed framework experiments show a significant improvement in the prediction of inaccurate resources of fog nodes by having a minimum delay, low processing time, improved accuracy, and the failure rate of prediction was faster in comparison to traditional LSTM, Support Vector Machines (SVM), and Logistic Regression.

## 1. Introduction

In recent years, fog computing has been introduced as a computing paradigm, bringing services, applications, and computation closer to consumers, and providing a good foundation for the Internet of Things (IoT). With the rapid growth of the Internet of Things (IoT), there is a drastic shift from the current Internet into an interconnected network, which is reshaping present and future computing paradigms. Rather than only collecting information from the environment, the era is also becoming interactive with the physical world to provide services such as information transfer, analytics, and communication [1]. IoT devices typically process huge amounts of data in the cloud, providing infinite computing, networking, and storage capabilities [2]. The cloud, however, has several downsides, including high bandwidth, latency, and resource management costs. As an example, an application in a smart mobility context such as a traffic monitoring system and emergency response system should not be tolerant of delay and latency, which will result in a lot of data exchange between the application and the cloud [3]. To push the main cloud innovations like virtualization, manageability, storage, and network resources into the edge of the network, keeping the deployment of applications and services closer to consumers, and mitigating the main issues between cloud and IoT [4,5], fog computing has emerged as a trend [6]. At present, in the area of IoT devices, reliability should receive considerable attention in the era of fog computing. In fog computing studies, several fog nodes serve tasks to deliver high-availability services to IoT devices. The fog devices are composed of hardware components such as hard disks, memory modules, network cards, and processors. There is always the possibility of failure of insufficient resources, and capacity for each of those. A device’s failure rate during its lifetime (typically 2–4 years in the industry) is quite low, but the numbers can increase. Due to its size, it is not appropriate to consider it an exception, as it would cause consumers to suffer performance degradation [7]. By analyzing these devices’ failures or predicted failures, one can consider an equipped system that can endure failures and reduce costs [8]. The failure of fog devices by having insufficient resource constraints may result in performance degradation for end users as services would not be available. We must understand how failures are predicted and how they are caused to improve tolerance for failure. Furthermore, a model is required that proactively predicts that resources are insufficient and moves data and workload to another fog node in advance to prevent service disruptions. This research work focuses on predicting fog node failure due to deficient resources while executing IoT applications in the fog environment.

To proactively predict the unsatisfactory nodes, a Recurrent Neural Network can be used to generate a sequence and label it. In addition to the current state, the previous state also determines the state of the network. In traditional RNNs, input information can only be stored for a short period, limiting their ability to model long-range input sequences [9]. As an alternative to traditional RNNs, the LSTM architecture is designed to improve information storage and retrieval. It has been demonstrated that LSTM models can be applied to a variety of tasks, including sequence handling, handwriting recognition, and sentiment analysis [10]. To model the dynamic nature of fog nodes, we can use LSTM-based prediction networks to predict the failure dependencies of computing fog systems. When there is a failure of the IoT application running on the fog device due to insufficient resources it could lead to a disaster of the application or shutdown of the fog device. To maintain the workflow at normal tendency and to keep the applications running, it is important to understand the causes of the outage, which gives the necessity for a rule-based learning approach on a deep neural network. The advantage of using a rule-based policy on a deep neural network is for further refinement such that the rules can be generated for the fog nodes that belong to the item set, which lowers the time complexity to help in the prediction of the failure of the insufficient resource. To determine accurate prediction, it is important to create rules based on relationships between the cause and type of insufficiency of resources of machine failure [11].

The main goal of this paper is to understand the importance of the prediction of the type of resource failure that occurs when there are inadequate resources in the fog nodes. If a small set of IoT applications are serviced by the fog nodes, and when there is an abrupt outage of fog devices due to the outburst of IoT applications, the fog nodes should be able to proactively predict the insufficiency of its resources and monitor the failure of the devices by the application placement into appropriate fog devices. The results from our study show that proposing a conceptual framework that combines the deep neural network properties along with the proposed rule-based approach determines the failure of the resource that is inadequate. Moreover, results on the performance of this approach show that it has a minimum delay, processing improves the accuracy and minimizes error with better failure prediction.

The remaining paper is organized as follows. Section 2 discusses the related works in various recurrent neural networks and rule-based approaches in a distributed computing environment. Section 3 gives the problem definition. Section 4 discusses the proposed methodology, which consists of the two network-based layers comprising of LSTM and CRP network policy and its components and algorithms in detail. Section 5 discusses the experimental setup, and various metrics used for the evaluation of the proposed approach, and the results are presented. Section 6 discusses the conclusion and how this study and research can be used in future work.

## 2. Literature Survey

There are two main categories for faults: processor view and generic view. Faults are divided into three groups from a processor’s perspective: crash, fail-stop, and Byzantine. These are primarily employed when a machine or resource fails. In the crash malfunction model, the CPU abruptly terminates operation at a specific point. When a failure occurs, the contents in the fail-stop are lost and cannot be recovered. Byzantine defects emerge as a result of an unanticipated failure, such as ageing or exterior damage to the infrastructure. These flaws are present in all processors and messages. There are three different kinds of generic view faults: transitory, intermittent, and permanent [12]. Transient issues only affect the current task execution and are fixed by restarting or rerunning the process. Rarely, intermittent malfunctions do occur. Permanent defects are flaws whose consequences cannot be compensated for. The following can experience faults in a distributed surrounding: operating system, user, middleware, hardware, task, and process flow [13]. Network difficulties, computer crashes, memory issues, and errors such as file not found, file staging, authentication problems, uncaught exceptions, problems related to data movement, and customer exceptions are a few of the prominent flaws that can arise [14].

For fog and certain other distributed systems, fault prediction and tolerance strategies are investigated. These strategies are divided into two categories: proactive and reactive models [15]. Included in the proactive model are:

- *Self-healing*: is defined as a system’s capacity to have a self-recovery mechanism for errors by employing particular fault recovery techniques on occasion procedures that involve tasks for monitoring.
- *Pre-emptive migration*: is described as a system’s capacity to transfer computation away from hazardous processing nodes in a proactive fashion.
- *System rejuvenation*: this is a procedure for regularly taking a system backup. Following every backup, the device is cleaned before the backup is restored, resulting in a refreshed state of the system.
- *Load balancing*: is employed to distribute the load on the processor and memory when it has reached its maximum limit. The workload of a CPU that has reached its maximum capacity is moved to a different CPU that has the processor and memory available.

The components of a reactive model are:

- *Checkpoint restart*: this function periodically saves the states of a task’s execution. In the event of a failure, the job is restarted from the most recent state that has been saved as opposed to starting from scratch.
- *Job migration*: in the event of a resource failure, the job switches to another instance of a similar and appropriate resource.
- *Replication:* used to produce numerous copies of jobs and store copies in various places, such as the primary backup strategy, which places the primary replica on one machine and the copy of the backup on a different device.

Numerous methods have been used to attain fault prediction based on these techniques. In the fault tolerance reactive approach, faults are dealt with after they have occurred. System maintenance strategies reduce the effects of occurring faults. Reactive strategies operate more based on response than on prediction. There are two main fault tolerance strategies used in distributed scheduling: checkpointing and duplication [16]. Reactive strategies are often conservative and do not call for system behavior analysis. As a result, they do not add any extra burden. As the term suggests, the proactive approach means that the system tends to be in a controlled state, prepared state, or managing any potential interruptions such as errors, faults, mistakes, and failures before they happen. In proactive approaches, the system condition is constantly monitored, and artificial intelligence algorithms are used to estimate the fault occurrence. Then, the necessary steps are taken to stop the fault from happening. These methods function based on prediction and experiences.

Many authors have recommended and put fault prediction algorithms into practice when faults are distributed. The author of [17] suggests a methodology for proactive failure prediction to predict device failure. They obtained a degree of accuracy for forecasting failures that vary from 70% online to 74% offline using supervised learning algorithms. The author of [18] suggests Fault-Tolerant Scheduling Method (FTSM) for Fog-Cloud environments. The approach uses a method where time-permissive demands are sent to the cloud, while time-responsive demands are sent to the edge devices. Based on the devices’ operational time between failures, FTSM determines the checkpoint duration. However, the authors did not consider any failure prediction for devices that rely on the varying availability of processing resources in fog devices. A Heuristic Fast Failure Recovery (HFFR) approach for defining software services that use function chaining in the environment of fog computing with failure examination is proposed by the authors in [19]. However, HFFR failed to account for the continuous changes in the resources that were available. In [20], the author proposed an effective resource-tracking service strategy and suggested that, in the fog environment, effective resource management requires effective failure handling. Reactions are executed in this case after the service for the request has begun. In this way, the fog devices’ status is regularly checked to look for malfunctions. Checkpoints, resubmitting, and replications could all be used for reactions [21]. Although several fault-tolerant approaches have been described for cloud [22] and grid computing [23], fault prediction for deficiency of resources in fog computing environments remains a challenge, and there is minimal research that contemplates it [24]. The authors of [25] have provided a fault-tolerant approach based on checkpoints that reduce the amount of storage space required to maintain checkpoints. Only the updated parameter values are saved using their mechanism.

For lengthy tasks, the authors of [26] propose a checkpoint-based technique that relies on allocating priorities to tasks. The authors of [27] have created a scheduling method that takes migration and checkpoints into account to handle failures. Ref. [28] proposes a checkpoint technique for fault prediction that uses hash tables and distributes the information from checkpoints. Four stages are suggested by the authors in [29] as a procedure for managing failures. Their method employs message logging and checkpoints to preserve the service states. Following that, it investigates the surroundings to learn more and flags errors. If a malfunction is anticipated, the protocol can make the proper choice to stop it. The protocol alerts the reliant entities to take reconfiguration measures in the event of failure. The protocol also chooses the appropriate steps for recuperation. A linear programming technique is used in [30] to assess the application of proactive and reactive recovery techniques. Their objective is to tolerate failures in a single commodity. Ref. [31] suggested using machine learning and sensor analysis of the data to forecast the failure of the device. When utilized for the analysis of sensor data in industrial automation, machine learning can allow better maintenance, such as failure diagnostics and preventative analysis of the device. Using industry-level sensors that are more accurate, durable, and robust is one simple way to address data quality problems, even though elements that lead to errors and affect sensor data quality are well understood. Applications that call for the creation of massive and dense sensor networks, including many IoT applications, are not possible with them. Most existing predictive models for machine failure focus on a single type of machine failure [32]. These models are difficult to apply to real enterprise manufacturing processes. The best-known specific method of rule-based learning is the Decision Tree algorithm. However, due to the fragmentation, the rules become prohibitively long and complicated [33].

In summary, prior failure management strategies in fog computing did not adequately account for the dynamic availability of fog resources. In this work, we propose an approach to proactively predict the failure of inadequate resources in the devices of fog while executing the IoT application. This research was carried out to propose a framework to predict a proactive solution to determine the dynamic resources when they are scarce in fog devices.

## 3. Problem Definition

Given a fog node or a set of fog nodes [*f_n_,ᵠf_n_*] for a fog computing environment and a collection of logs from this environment, let the probability of failure be *p_f_*(*W*)** occurring at the node within time window *W* when the resources of computation, storage, and bandwidth are inadequate. The data collected consist of features extracted from various fog nodes at different timestamps and failure labels provided by the administrator of the system. The solution is not specific to the type of failure but targets a general abnormal hardware malfunction when the resources are scarce. The objective of predicting failures is to alert the fog node failure before it occurs. This is of utmost importance as it gives the system administrator sufficient time to deal with the problem before it occurs. It is important to consider the time of early morning for evaluating the quality of predictive modeling defined by the predictive periods.

- *Predictive Period:* This period is a pre-defined period just before a failure. When an alert is given during this period there is enough time for the administrators to react and terms to be successful. The time the alert is given to the starting time of the failure is the specified time window W.

To overcome this problem, two approaches are carried out, the first one is the binary classification problem for predicting the failure of a fog node within a time window W before the occurrence of failure and the second approach is the rule-based policy approach. The model takes as input a set of input sequences as features and a target, which is a binary vector taking two complementary values that represent negative samples during the normal duration and positive samples during the predictive duration. If there is an alert reported during the predictive period before the failure, then the prediction is successful. The output from the model is essentially an alert probability and reported if such a probability exceeds a pre-defined threshold.

## 4. Proposed Methodology

**Figure 1:**
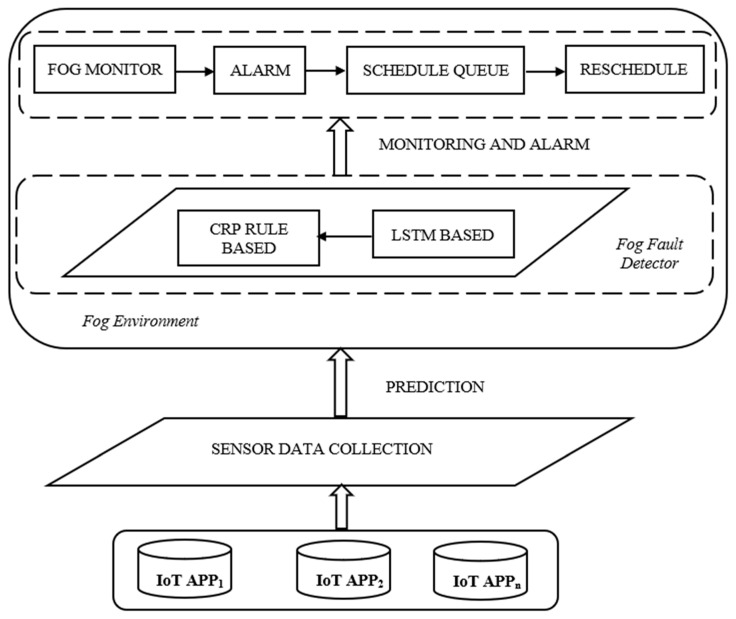
Overall architecture of the LSTM + CRP proposed framework.

The proposed prediction model is based on the dynamic failure of resources of fog nodes working on a two-composition layer. The first layer identifies the failure of the fog nodes, and the second layer identifies the insufficient resource of the fog node. The first layer works on the proposed LSTM for the prediction of final labels where the neural network output depends on the current inputs, their weights, count of the weights, and values of the previous neurons. LSTM structure has unparalleled natural advantages in extracting features. The biggest feature of the LSTM model is to allow memory operations to quickly learn useful features and filter out other unserviceable features. The prediction model outputs the probability of a failure, which is important in order to have temporal dynamics of the fog node. The second layer is the rule-based policy, which takes advantage of the context memorization operation of LSTM. The proposed rule-based policy is based on knowledge extraction of systems to identify the feature of resources in the inadequate fog nodes. It is further granulated to identify the resource in the fog node such as processing, memory, power, bandwidth, and availability based on CRP network policy. The overall architecture of the proposed methodology is given in Figure 1 and is described as follows.

The proposed overall architecture is described as follows. The front end consists of the IoT applications which are responsible for sensing and collecting the data and sending it to the fog devices for processing. The major portion of the fog devices in this work consists of the fault detector, which is designed to be proactive in nature. The fault detector consists of two major portions, the initial one is based on the LSTM Prediction network and the latter approach works on a proposed CRP rule-based approach. The fault detector works with the data from the fog device during its operation. The results of fault detection from the fault detector are transmitted to the fault monitor that visualizes the condition of the fog device and predicts the warning if there are any insufficient resources. The back end takes all the collected data from the fault detector to develop the model for fault detection. The prediction results are sent to the fault monitor, which is responsible for raising an alarm by taking necessary actions like rescheduling the fog devices or rebooting.

### 4.1. Fault Detector

The fault detector that is programmed into the fog device collects various data from the fog device such as MIPS, RAM, bandwidth, uplink, downlink level, and power during the operation of the fog device. The database of this detector is set up to allow for quick storing of acquired data and analysis findings. The data in the database can be used to train the fault detection and prediction algorithms in the back end. Each layer is discussed in detail as follows.

#### 4.1.1. LSTM Fault Prediction Network

**Figure 2:**
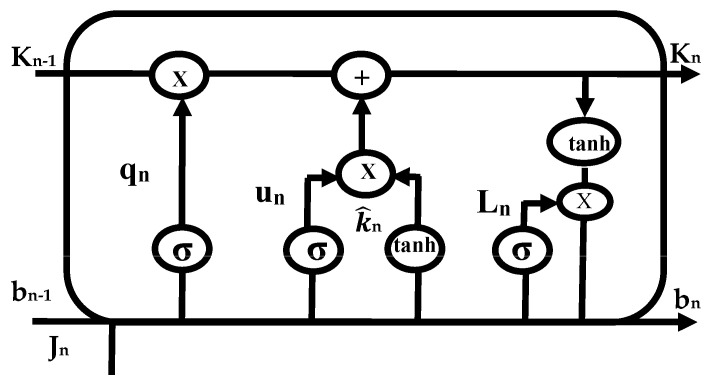
LSTM cell.

The predictive neural network architecture is represented in Figure 2. Given an input feature sequence, which is a vector x=xt−L+1,……. xt, with the length of the sequence being *L*, which is a long historic sequence of arbitrary length passed to a stack of multiple hidden layers that are recurrently connected through weighted connections that compute the hidden vector sequences h=ht−L+1,……. ht with output sequence vector y=yt−L+1. The output vector parameterizes the probability distribution *Pr *(*dt/yt*)** of the target dt. Unlike traditional RNNs, LSTM introduces a built memory cell for long-term dependencies [34] to store information on previous time steps.

**Figure 3:**
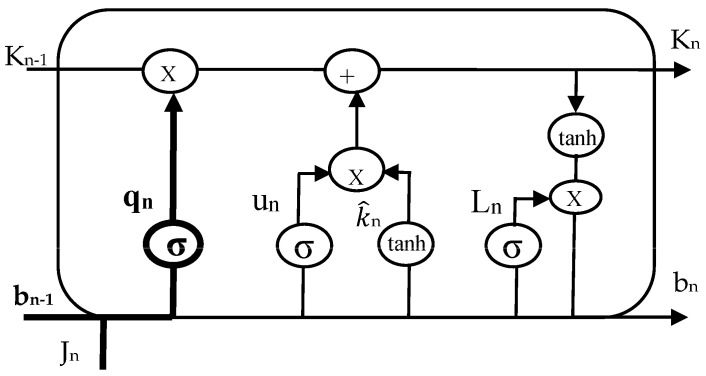
Forget gate of LSTM cell.

**Figure 4:**
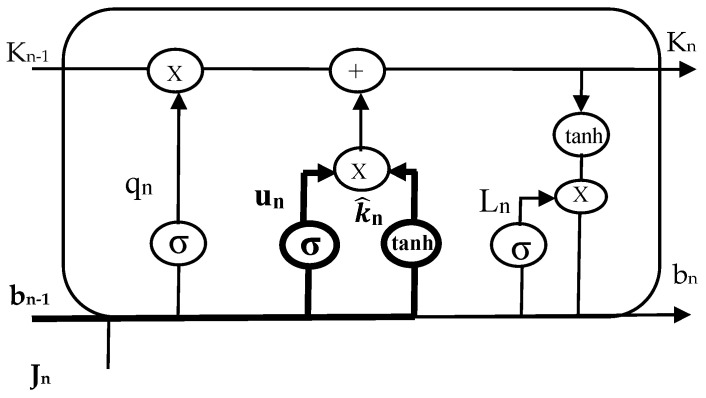
Input gate of LSTM cell.

**Figure 5:**
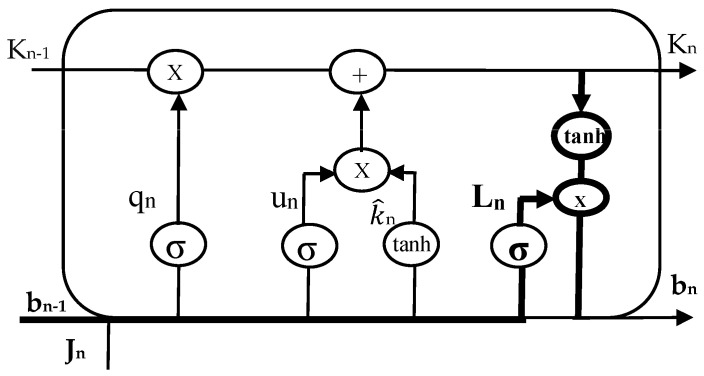
Output gate of LSTM cell.

The most efficient method for solving problems involving sequence prediction is thought to be LSTM networks. The ability of LSTM to memorize patterns for numerous sequences is its most significant feature. As a result, LSTM has an edge over traditional feed-forward RNN and NN, which are unable to accurately predict future values using past data. A predominant LSTM network has many blocks of memory, also known as cells. As each cell progresses, it transfers two states to the next cell. Algorithm 1 describes the hidden and cell states. Memory blocks are responsible for remembering information. The three main gates control how this memory is manipulated. LSTM cells are illustrated in Figure 3, Figure 4 and Figure 5, such as forget, input, and output gates along with the roles they play and their representations.
**Algorithm 1:** Computation algorithm of LSTM Cell***Input:***   $J_{n}$
***Output:*** $l_{n}$
***Generating Algorithm:***
***BEGIN***
***Initializations*** $b_{n-1}$, $C_{q}$, $C_{u}$, $C_{l}$, $x_{q}$, $x_{u}$, $x_{l}$**while** i < time_steps: **do**  **Step 1: LSTM—Forget gate**    $q_{n}=\sigma \left(C_{q}\times \left[b_{n-1},J_{n}\right]+x_{q}\right)$  **Step 2: LSTM—Input gate**  $u_{n}=\sigma \left(C_{u}\times \left[b_{n-1},J_{n}\right]+x_{u}\right)$ ${\hat{K}}_{n}=tanh\left(C_{k}\times \left[b_{n-1},J_{n}\right]+x_{k}\right)$ **Step 3: LSTM—Output gate** $l_{n}=\sigma \left(C_{l}\times \left[b_{n-1},J_{n}\right]+x_{l}\right)$ $b_{n}=l_{n}\times tanh\left(K_{n}\right)$**end while** ***END***

- *Forget Gate:* This gate, which is the first one in the LSTM cell, determines whether or not the data from the preceding stamp will be kept. The data from the current input state *J_n_* and hidden state *b_n_*_−1_ are acquired, a function named sigmoid is applied to produce an output within 0 and 1, and then the cell state from the preceding timestamp is multiplied with the result. If the decisive number is 1, nothing is forgotten. However, if the decisive number is 0, everything is forgotten.
- *Input Gate:* A value within 0 and 1 is produced by applying another function of sigmoid to the current *J_n_* and hidden *b_n_*_−1_ states in the input gate before the tanh function is used on it. The state of the cell is then modified to a different cell state after taking the vector input values and adding them stepwise.
- *Output Gate*: The information contained in the hidden state for the following cycle is determined by applying a third function of sigmoid to the current state, the preceding hidden state, and the recent state of the cell produced in the input gate. Point-by-point multiplication is performed on both outputs and chooses what data the subsequent cycle’s hidden state will contain. The hidden state is transmitted over to the following step together with the new state of the cell.

##### LSTM Fault Trainer

At the middle end of the development platform for fault model detection, the trainer is built using LSTM. Based on the gathered data, it attempts to determine the appropriate model to categorize the fault or normal state of the device in fog. Using time series information, LSTM is used to predict fog device failure. By configuring the various gates such as input, forget, and output, LSTM avoids the problem of vanishing gradient, which arises when the network weights in the time series data have not been updated properly [35,36]. It tries to find the right model by classifying fog device statuses into normal and faults. The information from the previous cell should be erased according to the forget gate’s settings. The input gate designates the data that will be given to the input’s current value. The amount of data that has to be conveyed from the cell is determined by the output gate. With the use of these gates, a desirable result can be created by holding onto crucial data for a long time while discarding irrelevant information [37]. The tuning of the hyperparameter is crucial for the creation of an effective LSTM model. It is necessary to determine various hyperparameters, including the dropout rate, the optimizer employed, the degree of regularization in the kernel, and the reduction of hidden state dimensionality. The first two parameters are crucial for LSTM model training, while the final two help prevent overfitting.

An optimizer such as RMSprop, Adam, or AdaGrad can be factored in for appropriate optimization to find the overall optimum loss. It is vital to regulate the learning rate and monitor the error that it needs to minimize throughout the learning period to prevent the local optimum. The weight decay method assigns penalties that correlate to large weights indicated by regularization to prevent the overfitting issue. The dropout approach permits certain weights to be neglected at random throughout the training phase depending on the dropout rate, which is also widely known for reducing overfitting. The strategy can reduce overfitting while improving the representation of training data. Due to the characteristics of the training data and the variety of neural network topologies, finding the optimum hyperparameters requires time. Therefore, it is frequently easy to find the best combinations of hyperparameters utilizing grid or random search approaches. In this study, the best hyperparameters are found using the grid search strategy. The fog data, which use time series with a specified length and binary machine status data such as a fault or normal state are fed to the LSTM systems’ input nodes and a singular node output, accordingly, to train the fault prediction model based on LSTM.

Here, the state of the machine is used to determine the outcome of the operation as the desired outcome value and the full sequential vibration information of every operation is sent into the vector input. The function based on sigmoid was used as a single node’s output activation function to build the machine binary state, and a binary function established on cross-entropy was used as the model’s function loss to adjust the weights depending on the divergence between the expected and actual output values. Algorithm 2 works on the above-discussed approach and is illustrated as follows. Table 1 indicates the notations used in this paper.
**Algorithm 2:** LSTM Fault Trainer***Input:*** Total no. of fog devices-*α* Each fog device—*β* Resources of fog devices—{FN_C+S, FNA_C+S, FN_UD, FN_level, FN_pow} No. of features, *fNum* Function for Transformation-*g(.)–{w1,w2}****Output:*** Running status of the device(fog status)—*ψ****Generating Algorithm***     ***BEGIN***     G = []     *lt = α [len (α) – 1]*    for each *β* in *α*       while *j* < *fNum* *           m⟵m + pow (β [j] – lt[j], 2.0)* *          j + 1*       end while           G.append(sqrt (*m*))    end for        // Standardizing the values of G to [− 1, 1]            *G⟵std_normalize(G)*           while *j* < len (*α*)            *E[j]⟵g(j)*             *j⟵ j + 1*           end while           *E⟵std_normalize(E)*           while *j* < len(*α*)            ψ [j]⟵w1* G[j] + w2* E[j]           end while         return *ψ*    ***END***

#### 4.1.2. CRP Rule-Based Network Policy

**Figure 6:**
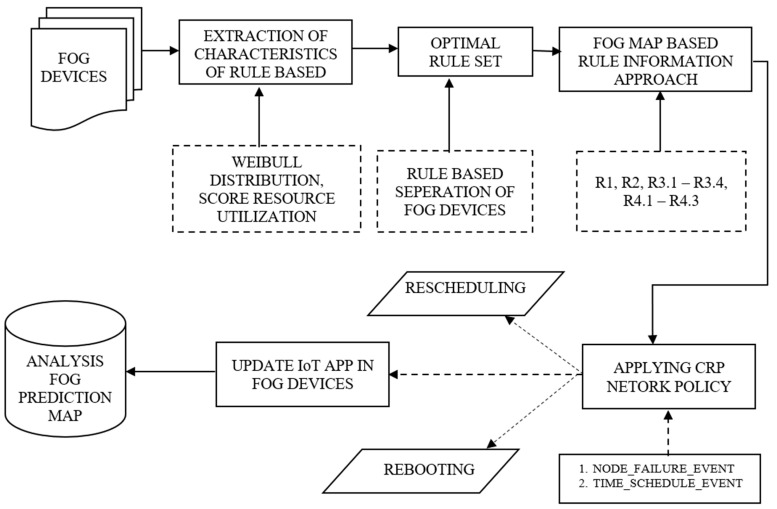
Architecture of CRP rule-based network.

A CRP rule-based network policy is key to dealing with the prediction of fog node failure concerning the specific resources of MIPS, RAM, and power and to avoid the fog node going into an idle state or the application processing going into an unprecedented waiting queue. If necessary, once the failures are predicted, the workload of the applications can be pushed into future scheduled nodes available in the scheduled queue. A rule-based resource manager is proposed for successfully identifying the specific fog resources for the IoT applications and data that are inadequate. With the help of the resource manager, prediction of failure of fog nodes can be done proactively such that the fog nodes can be rescheduled or scaled up to allocate resources on demand. The goal is to identify all frequently occurring resources and the relationships between them. With the significant increase in data volume from the IoT applications and the demand for the establishment of the association with the fog nodes, rules are extracted to predict the insufficient resources of fog nodes to avoid the processing delay of the IoT applications in the fog devices. The architecture of the CRP rule-based network policy is given in Figure 6. The failure traces of fog nodes have been generated as follows.

(a) We horizontally divide the resources of each fog device in the data set.
(b) Each node consists of its scanned data subset that generates a set of resources.
(c) The resources of each fog device are divided into r partitions that are different.
(d) These r partitions of the nodes accumulate the score of the fog device and produce the final score, which determines the failure after comparing it with the minimum score.
(e) From the observed outputs, the set of failed failure traces of fog nodes is generated.

##### Characteristics of Rule-Based Aspect Extraction Approach

The characteristics of the proposed rule-based policy to predict the failure of the resources of the fog devices are dynamically proposed based on resource availability. The key difference in this policy is that in this rule-based approach, the nodes are arranged to be scheduled in the order of their last failure times. The failure rate follows a Weibull distribution with the parameter of the shape taking less than 1. The nodes that are suspicious of failure and the longer the node is available, the node becomes more robust. Such nodes are put in the head (front) and during the request, the nodes in the tail (rear) are provided. The failure rate is defined as the conditional likelihood of a system that failed between timestamp *k* and timestamp *k +* Δ*k*, taking into consideration that it did not fail at timestamp *k* [38]. Because the rate of failure is a function of timestamp *k*, it is designated as the *λ*(*k*)** named as the rate of failure function. This function evaluates a single node’s dependability based on its uptime from its most recent reboot to timestamp *k*. The rate of failure is defined in Equations (1) and (2) as follows:

(1) $$\lambda \left(k\right)=lim_{\Delta t\to 0}\frac{P\left(k<K\leq k+\Delta k|K>t\right)}{\Delta t}\;$$

(2) $$\lambda \left(k\right)=\frac{pf\left(k\right)}{1-cf\left(k\right)}\;$$

where the probability density function is given as *pf* and the cumulative distribution function is termed as *cf*. Researchers have essentially researched a variety of service systems [39,40,41] and found that Internet services and high-performance computing seem to have various characteristics of failures in the system. System logs failure in event traces have been studied by researchers in [39,40] and identified the time concerning failures such as reboot node failure. This stochastic process is predicted to follow the Weibull shape (*sh*) distribution and Weibull scale (*sc*) distribution with a parameter of shape < 1. Weibull (*sc*, *sh*), *pf*(*k*)** and *cf*(*k*)** are given in Equations (3) and (4), respectively. Therefore, the rate of failure of the Weibull scale and Weibull shape is computed in Equations (5) and (6).

(3) $$pf\left(k\right)=\left(\frac{sh}{sc}\right)*{\left(\frac{k}{sc}\right)}^{sh-1}*e^{-\left(\frac{k}{sc}\right)sh}\;$$

(4) $$cf\left(k\right)=i-\left(e^{-\left(\frac{k}{sc}\right)sh}\right)\;$$

(5) $$\lambda \left(k\right)=\frac{pf\left(k\right)}{1-cf\left(k\right)}\;$$

(6) $$\lambda \left(k\right)=\left(\frac{sh}{sc}\right)*{\left(k/sc\right)}^{sh-1}\;$$

i.e., if there are two nodes, namely *Xnode* and *Ynode*, with their uptimes given as *X_uptime* and *Y_uptime* with *X_uptime* being greater than *Y_uptime*. Their rates of failure are *λX = λ*(*X_uptime*)** and *λY = λ*(*Y_uptime*)**. Hence, when *sh* < 1, *λX* < *YY*, i.e., compared to *Ynode*, *Xnode* is more reliable or less vulnerable to failure, i.e., the node that just failed is vulnerable to other failures, but if it keeps running continuously for a while, it will become more resilient. This failure node characteristic suggests that taking the most recent failed node for a certain duration of reliability evaluation is useful [40].

The score method is defined to take into consideration how to dynamically allocate an IoT application to a fog node; the dynamic allocation and the failure of the fog node depend on the weighted sum of the resources. If there are *k* fog nodes, then the number of nodes executing the IoT application *h*, is *k* ≥ 4 h. The distribution of fog nodes and their resources *k* are expressed in Equation (7). *α_k_* expresses the ratio of the distribution of resources of the fog node k given in Equation (8), ψ expresses the score of the overall capability of the fog node executing the given IoT application given in Equation (9), A expresses the sum of the resources of the IoT application given in Equation (10).

(7) $$\;\beta_{k}\;≅\;\alpha_{k}\times \left|A\right|$$

(8) $$\alpha_{k}=R_{k}÷R$$

(9) $$\psi =\sum_{k=1}^{n}R_{k}$$

(10) $$A=\sum_{i=1}^{k}A_{i}\;$$

A fog node’s integrated computing capabilities in the fog environment are tied to four resource configurations: CPU speed and utilization, accessible memory size, bandwidth uplink/downlink speed, and the node k’s integrated computing capability weight, which are defined in Equation (11) as follows.

(11) $$\psi {\;}_{k=R_{k}^{UD}+R_{k}^{CPU}+R_{k\;}^{RAM}+R_{k}^{POW}\;}$$

where, the weight of uplink and downlink is $R_{k}^{UD}=g_{k}/g$, weight of CPU node k is $R_{k}^{CPU}=l_{k}/l$, weight of memory is $R_{k}^{RAM}=$ $c_{k}/c$ and weight of power is $R_{k}^{POW}=p_{k}/p$. The ratio among these four resources of the node and the associated points of reference resources illustrates the score performance measure deviation of nodes that are heterogeneous. Each node’s computing resources have diverse effects on dissimilar forms of loads, resulting in a relationship between the integrated weight and the load type. Equation (12) determines the node’s weighted load capacity,

(12) $$R_{k=\lambda_{k}^{1}\times R_{k}^{UD}+\lambda_{k}^{2}\times R_{k}^{CPU}+\lambda_{k}^{3}\times R_{k}^{RAM}+\lambda_{k}^{4}\times R_{k}^{RAM}}\;$$

where the parameter $\lambda_{k}^{p}$ signifies the dynamic ratio of resource utilization of UD, CPU, RAM, Power of node k by the load, and $\lambda_{k}^{1}+\lambda_{k}^{2}+\lambda_{k}^{3}+\lambda_{k}^{4}=1$.

##### Optimal Rule Set and its Robustness

To predict proactive failure based on rules generated over the proposed LSTM network. The parameters considered to generate the rules are determined by the position where the fog nodes are placed from the IoT devices along with the dependency relationship of the infrastructure of the fog nodes to the requirements of the IoT application taking into consideration the CPU, RAM, the uplink, downlink, and level denoting the resources and position of the fog node in the infrastructure. The power indicates the capability of the fog node when it approaches the consumption of being busy and idle. If either of these parameters do not satisfy the given rule, then the resources are insufficient. The following rules are taken into consideration to identify the failure traces of fog nodes.

(a) Rule 1:

Rule 1 is defined as follows, where R1 depends on the computational resources and storage of fog nodes. If the resources of CPU and RAM are less than that of the placed IoT application, then there is inadequate CPU and RAM. If either of these parameters does not satisfy the given rule, then the resources are insufficient. If the resources in fog nodes are evaluated by either link, power, or level, then it returns a score between −1 and +1, ‘‘Insufficient resources”.
R1 = R1(FNA_C+S)
R1: if res(FNA_C+S v FN_UD v FN_pow v FN_level) < res(Y_C+S) →ψ (−1,+1), {insufficient resources of CPU + RAM}

(b) Rule 2:

Rule 2 is defined as follows, where R2 depends on all the parameters of the resources of the fog based on CPU, RAM, uplink, and downlink bandwidth, and the placement of the level of the fog node along with the aspect of power. If any of the given resources are insufficient, then insufficient resources are present. If the resources in fog are not evaluated by all the parameters of CPU, RAM, link, power, and level, then it returns a score between −2 and +2, indicating ‘‘Insufficient resource”. Such nodes will be removed from the scheduled queue.
R2 = R2(FNA_C+S, FN_UD, FN_pow, FN_level)
R2: if res(FNA_C+S ᴧ FN_UD ᴧ FN_pow ᴧ FN_level) < res(Y_C+S) →ψ (−2,+2), {FN_fault},{del(FN)}

(c) Rule 3:

Rule 3 is defined as follows, where R3 depends on the process of if there is an available resource of CPU or RAM or if both are present, which is nearly inequivalent to the resources demanded by the IoT application; then the score of the aspect changes as follows.

*R3.1:* Strong availability of resources: If available resources are greater than the resources required by IoT applications, then it has a strong effect on the score and indicates “strong sufficient resources”.

*R3.2:* If score (CPU, RAM) < 0 and available resources do not belong to a certain level, then score (CPU, RAM) < score (available resources in IoT) then insufficient resources.

*R3.3*: If score (CPU) < 0 and score (RAM) > 0 and available resources belong to a certain level then score (CPU, RAM) < score (available resources in IoT) then insufficient resources of CPU.

*R3.4*: If score (RAM) < 0 and score (CPU) > 0 and available resources are at a certain level then score (CPU, RAM) < score (available resources in IoT) then insufficient resources of RAM.
R3 = R3(FN_C+S)
 R3: if avail(FNA_C+S)! = res(Y_C+S) if avail(FNA_C+S) > res(Y_C+S) →ψ  = True        {strong sufficient resources} if avail(score(FNA_C+S)) > 0 ᴧ avail(FN_level) >= 1 →    ψ (FNA_C+S) < ψ avail(Y_C+S)      {insufficient resources of CPU, RAM and level} if avail(*ψ* (FN_C)) < 0 ᴧ avail(*ψ* (FN_S)) > 0    ᴧ avail(FN_level) >= 1 ->     ψ (FNA_C+S) < ψ (Y_C+S)     {insufficient resources of CPU} if avail(ψ 
(FN_S)) < 0 ᴧ avail(ψ (FN_C)) > 0     ᴧ avail(FN_level) >= 1 →    ψ (FNA_C+S) < ψ (Y_C+S) then  {insufficient resources of RAM}

(d) Rule 4:

Rule 4 is defined as follows, where R4 depends on the node based on power consumed during the idle and busy states; then the score will be updated as follows,

*R4.1:* If score (FN_pow) > 0 and available resources belong to a certain level then score (FN_pow) > score (IoT_pow) then sufficient resources.

*R4.2:* If score (FN_pow) < 0 and available resources belong to a certain level then score (FN_pow) < score (IoT_pow) then insufficient resources of power.

*R4.3:* If score (pow_idle) < 0, then score (fn_busypow) = score IoT _power) then insufficient resources of idle power, fog node is busy.

R4 = R4(FN_pow)

R4: if res(FN_pow € (busy,idle)
   if *ψ* (FN_pow) > 0 ᴧ avail(*ψ* (FNA_C+S)) > 0 ᴧ    avail(FN_level) >= 1    ->    *ψ* (FN_pow) > *ψ* (IoT_pow)    {sufficient resources}   if *ψ* (FN_pow) < 0 ᴧ avail(*ψ* (FNA_C+S)) > 0 ᴧ    avail(FN_level) >= 1    ->    *ψ* (FN_pow) < *ψ* (IoT_pow)    {insufficient resources}     if *ψ* (pow_idle) < 0 ->     *ψ* (fn_busypow) = *ψ* (IoT_power)     {insufficient resources of power}

##### CRP Rule-Based Algorithm Policy

The rule-based policy that works along with LSTM is proposed based in an event-driven way. It is called whenever the resources of the fog nodes that are predicted by the LSTM model are insufficient. The events invoked are fog node failure and time schedule events. The fog node failure event indicates the failure of the fog node with respect to all the resources that are insufficient. The current time of failure is recorded, and the fog node is rescheduled by pushing it into the tail of the idle pool such that it is recovered over a period of time. A new node is pulled from the scheduled fog nodes to cater to the services of the running IoT applications. The time schedule event arises each time when the node was predicted to fail based on the optimal rule set generated.

If any of the rules are true predicting the precise insufficient resources of fog nodes, then that particular fog node is redirected to be rescheduled, pushing that fog node into the idle list and pulling out a new fog node based on the demand of the failure of the current fog node from the scheduled queue. If either of the rules are not satisfied and cannot be rescheduled, then the reboot of that node is required, and it must be placed in the scheduled queue. In case the high priority task is almost completed, and the node fails, then the task is moved to the cloud. If either of these conditions is not satisfied, and if a new failure of resources has been identified beside the rules generated, new rules are appended to the rules identified based on the classes observed from the training data. This is illustrated in Algorithm 3. These new rules that are generated are illustrated in Algorithm 4.
**Algorithm 3:** CRP Rule-Based Network Policy***Data_Structure:*** Fog_Node_List, a list of nodes that cater to IoT applications. Fog_Node_Pool_Schedule, a list of scheduled nodes waiting to service the IoT Applications Node_Pool_List, idle nodes list. In this list, the most recently failed nodes are moved to the top and they are set to the current time. In the meantime, move non-failed nodes, including those that are intentionally rejuvenated, to the end of the list. HPC_IoTApp_Running_List, HPC running IoT Applications in a list. HPC_IoTApp_Finished_List, HPC finished IoT Applications in a list***Output:*** Prediction of Failure***Assumptions:*** All of this data is extracted from the logger of the devices Fog_Node_failure_Event; Time_Schedule_Event;***Generating Algorithm:*** ***BEGIN*** while (RM_getevent(e_type)  // Resource Manager identifies the failure event  switch(e_type)    case Fog_Node_Failure_Event:      put FailureNode.uptime = CurrentTime;          push(Fog_Node -> Node_Pool_List)           pull(Fog_Node_Pool_Schedule)    case Time_Schedule_Event:       HPC_IoTApp_Running_List → request(res(Fog_Node))      for each Fog_Node in Fog_Node_List         if ({R1 or R2 or R3 or R4} == True)          Redirects(FogNode to Rescheduler)           push(Fog_Node -> Node_Pool_List)          push(HPC_IoTApp_Running_List -> Fog_Node_Pool_Schedule)           Allocate avail(FNA_C+S) -> avail(x_C+S)          Response.Redirect (FogNode);        end if        if ({R1 or R2 or R3 or R4} == False)          if(!(Rescheduled))              reboot()           elif(lowpriority)              push(HPC_IoTApp_Running_List -> cloud)          elif (new_rule)              generate_new_rule(new_rule)           end if        end if        for each IoTApp in HPC_IoTApp_Running_List           if(IoTApp.ExecuteTime + IoTApp.StartTime > CurrentTime)               put IoTApp.status = finished               push( IoTApp -> HPC_IoTApp_Finished_List)                push(Fog_Node -> Fog_Node_Pool_Schedule)           end if        end for      end for    end switch end while ***END***

**Algorithm 4:** Generate new rules***Input:*** S is the list of training data C is the list of different classes***Output:*** R Rules are produced, R = []//initially an empty list.***Generating Algorithm*** generate new_rule() Begin if (C ∄ [R1,R2,R3,R4])  // the new failure class found does not                    
belong to any of the rules.    for every class C      // for every new class found     while(C != S)   // while the class does not belong to                        training data       create new_rule ∈ C        R.append(new_rule)     end while    end for end if End 

### 4.2. Fault Monitor

The fog fault monitor is responsible for raising an alarm to the scheduler when the fog fault detector predicts the failure of insufficient resources using the conceptual framework of LSTM and CRP. The monitor is responsible for pulling the fog device from the running queue and place it in the idle queue to recover and move to the scheduled waiting queue. The monitor serves as a layer between the detector and the scheduler such that the IoT applications run effectively and are serviced by the fog nodes.

## 5. Results and Discussion

### 5.1. Experimental Setup and Failure Modelling

iFogSim toolkit is used for the simulation of Fog Computing scenarios. It provides basic classes for describing data centers, virtual machines, applications, users, computational resources, and policies for the management of diverse parts of the system. iFogSim is a simulation framework that supports seamless modelling and experimentation of fog computing infrastructure, including data centers on a single computer [42]. It has a virtualization engine, which assists in creating and managing multiple, independent, and co-hosted virtualized services on a data center node. It supports the performance evaluation of policies for resource provisioning and scheduling. The fog nodes having heterogeneous type resources are considered for simulation. Various fog devices have been created whose configurations are illustrated in Table 2 and the parameter settings of the IoT application has been illustrated in Table 3. There could be a situation where all the nodes that are scheduled are overloaded and there would be no sufficient resources, or the applications are allocated to fog nodes with insufficient resources at different intervals of time.

The following IoT applications running on fog nodes are used in car parking [43], smart waste management systems [44], and smart factory [45]. The pseudo-code of these applications is detailed in the following work [46]. Since there are no failure traces of fog nodes for the IoT applications, a failure traces dataset is created by considering the following IoT applications where the fog nodes that cater to these applications tend to become unreliable with the resources allocated at some instance of a time as the demand of the application varies. The traces are generated through iFogSim by monitoring the fog devices for the given IoT application. Each fog node consists of multiple components of resources with the possibility of various applications running on them. This was collected when the system failures were recorded by the system administrator.

The dataset for the traces of the fog devices was generated along with the characteristics of each node after running the IoT applications and is represented in Table 4. The failure takes into consideration multiple factors where resources were insufficient to run the IoT applications and could not function properly and is completely down. These traces are run on various algorithms of the proposed LSTM + CRP rule-based, LSTM, SVM, and logistic regression to determine which method is suitable to determine a proactive fault tolerant fog device. The performance of the proposed work is evaluated using the failure trace dataset, which has resources such as MIPS, memory, uplink, downlink bandwidth, level of the fog device, and busy and idle power, which are collected by running the IoT applications on various fog nodes for a period of ninety hours to obtain multiple resource utilization. The availability of CPU and RAM is determined by the remaining resources left when the IoT applications are running on fog devices.

The experiments are executed with varying sizes of fog nodes and IoT applications where fog nodes are allocated dynamically as per the demand of the IoT applications. In a real computing environment, there could be an outage or massive failure of physical servers, especially at peak hours causing overloads and resource contention. Accordingly, we consider a sudden peak of aggregated load (resource demand) of all fog devices, which is greater than available resource capacity, as a fog outage is predicted periodically.

### 5.2. Evaluation Metrics

All the performance metrics discussed are taken from the following work [47,48]. The proposed model’s performance evaluation is based on the following parameters: minimum delay, processing time, performance accuracy, error measures, and prediction of failure.

a. Minimum Delay

Minimum delay is defined as the time taken to predict the failure of fog devices. It is the time an IoT application was placed in a fog node and the time the execution of the IoT application stops due to insufficient resource execution. The calculation is given in Equation (13) as follows.

(13) $$\;delay_{k}^{n}=F_{sp}^{n}-A_{o}$$

where $delay_{k}^{n}$ is denoted as the delay for n IoT applications running of fog, which involves fog devices. $F_{sp}$ is the start time of IoT application execution and n—$A_{o}$ is the time an IoT application has stopped the execution in a fog node.

b. Processing Time

Processing the prediction of failure of insufficient resources for IoT applications requires computing time. The given Equation (14) can be used to calculate the amount of time that passes between the start and end time for predicting the computing capability of insufficient resources when the IoT application is processing in the fog devices.

(14) $$\;ct_{k}^{n}=c_{en}^{n}-c_{st}^{n}$$

where *n* is the fog device that is involved in IoT application and $ct_{k}$ is the computation time for prediction processing. $c_{st}^{n}$ is the start time and $c_{en}^{n}$ is the end time for predicting the capability of computation of insufficient resources.

c. Performance Accuracy and Error Measures

The R^2^ score that is specified as the degree of a dependent variable’s variance that can be predicted using the independent variable is the metric that is used to determine the proposed work accuracy. Equation (15) provides the following R^2^.

(15) $$\;R^{2}=1-\frac{\sum {\left(y_{i}-{\hat{y}}_{i}\right)}^{2}}{\sum {\left(y_{i}-{\bar{y}}_{i}\right)}^{2}}\;$$

(16) $$R_{adj}^{2}=1-\left[\frac{\left(1-R^{2}\right)\left(n-1\right)}{n-k-1}\right]\;$$

(17) $$Norm.RMSE=\frac{\;\sqrt{\frac{\sum {\left(\hat{y_{i}}-y_{i}\right)}^{2}}{n}}}{\;y_{i}max_{i}-y_{i}min_{i}}$$

The inclusion of needless variables decreases the Adjusted R^2^ score because it penalizes the use of independent variables towards prediction. R^2^ and adjusted R^2^ are never greater than one another. Equation (16) is utilized to determine the Adjusted R^2^_._ Normalized root mean square error (Norm. RMSE), a value between 0 and 1, where 0 indicates the most desirable value, is used to measure errors for the model’s performance. The normalized RMSE is calculated using the formula given in Equation (17). The number of parameters in a model is taken into account by calculating the residuals squared denoted as R^2^ where $y_{i}$ is the actual value of the fog node i, ${\hat{y}}_{i}$ is the predicted failure of fog node and ${\bar{y}}_{i}$ is the mean value of y. The Adjusted R^2^ is denoted as $R_{adj}^{2}$ where R is the residual square, n is the total number of fog nodes, and k is the number of resources of each fog node. $y_{i}max_{i}$ and $y_{i}min_{i}$ is the maximum and minimum value of each resource.

d. Failure Prediction

The prediction of failure of inadequate resources for the fog devices is given in Equations (18) and (19) and is used to apply the mean time before failure and the mean time to recover to compute the failure prediction.

(18) $$\mathrm{MTBF}=\int_{k_{1}}^{k_{2}}\left(\frac{\sum_{j=1}^{n}U\_T_{j}}{n\_f}\right)$$

(19) $$\mathrm{MTTR}=\int_{k_{1}}^{k_{2}}\left(\frac{\sum_{j=1}^{n}D\_T_{j}}{n\_f}\right)$$

(20) $$\mathrm{Ava}{\mathrm{Avgr}}_{\;}=\frac{\mathrm{MTBF}}{\mathrm{MTBF}+\mathrm{MTTR}}$$

Accordingly, availability average is computed using Equation (20), where n_f is the overall number of resource failures, $\overset{n}{\underset{j=1}{\sum }}U\_T_{j}$ is the overall uptime, and $\overset{n}{\underset{j=1}{\sum }}D\_T_{j}$ is the overall downtime of n fog devices running the applications of IoT experienced throughout a time period (*k*_1_,*k*_2_)

### 5.3. Evaluation and Inference

A scalable deep learning framework for python, Keras, is used to efficiently generate and train models. The training data consist of an input sequence and a target output. Relevant features are retrieved to build the trace data while taking into account IoT application events, resource utilization statistics, and limitations. The attributes of memory instructions per cycle (MIPS), RAM, uplink and downlink, level of placement of fog node, and power are all expressed as properties of a class in the input sequence. The desired output is the fog device’s termination as finish or fail. The LSTM prediction model is made up of dense layers in which the input sequence is transformed into an intermediate sequence, after which an average pooling changes the sequence into a single representation that is fed into the CRP policy to get the output of the insufficient resource. Five-fold cross-validation is used to train and test the model. To forecast failure, the LSTM + CRP model is compared to the baseline LSTM [49], SVM [50], and logistic regression [51] models.

(a) Prediction of Minimum Delay

**Figure 7:**
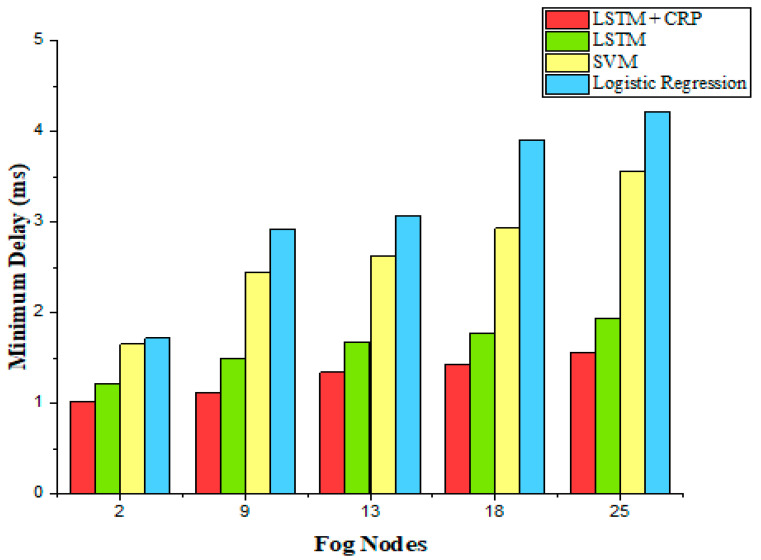
Prediction of minimum delay.

The given IoT applications were run on different fog devices having a range of 1 to 25 and the prediction of failure of different fog nodes was done based on the minimum delay given in Equation (13). It was observed that the prediction of failure was done with a minimum delay by LSTM + CRP in comparison to LSTM, SVM, and logistic regression, which seemed to predict a greater delay. As the fog nodes increased to 13, 18, and 25, the proposed LSTM + CRP model performed better than LSTM, SVM, and logistic regression by identifying the precise resource of the fog node that was determined as inadequate. The observations are illustrated in Figure 7.

(b) Processing Time

**Figure 8:**
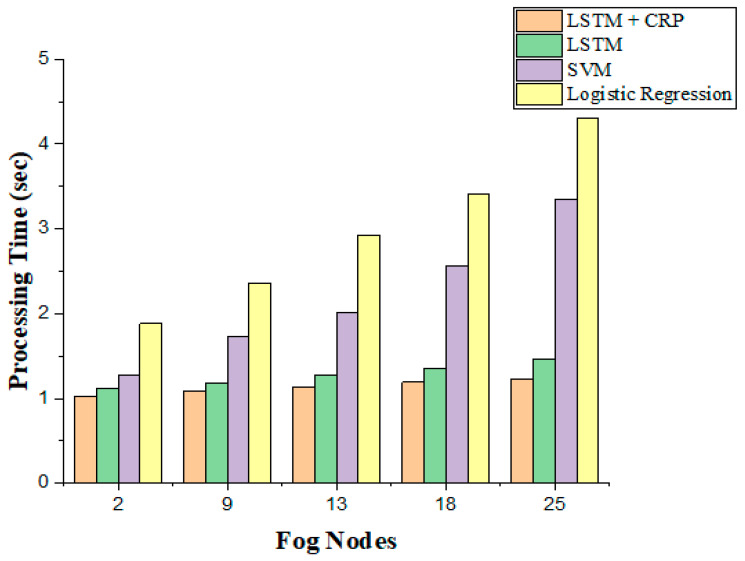
Minimum processing time.

The processing time to predict the failure of insufficient resources of fog nodes for the IoT applications given in Equation (14) is minimized when LSTM + CRP is applied when compared to LSTM, SVM, and logistic regression. As the number of fog nodes varies in the range of 2–25, it was observed that the proposed model of LSTM + CRP has faster computation in the prediction of insufficient resources than LSTM, SVM, and logistic regression models are capable of, as it only predicts the failure of the fog node. The observations are plotted in Figure 8.

(c) Accuracy and Error Measures

**Figure 9:**
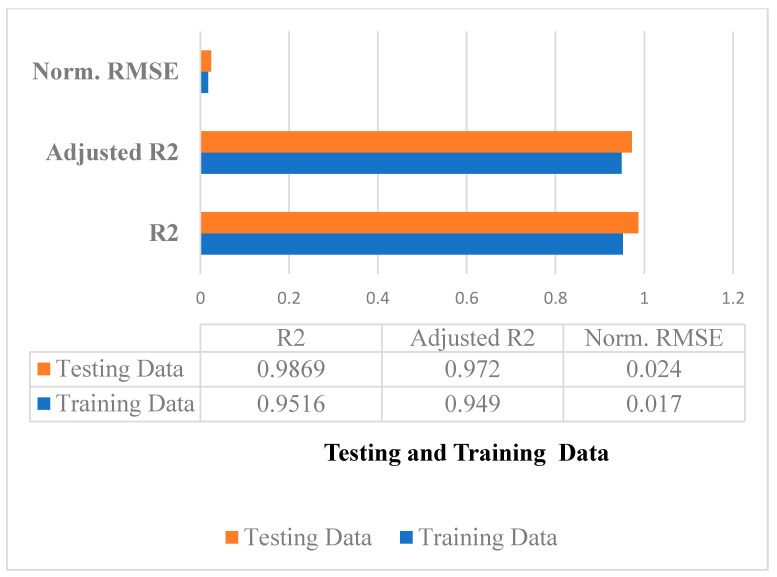
Performance metrics of LSTM + CRP.

For the proposed LSTM + CRP approach, LSTM network, SVM, and logistic regression, the performance metrics R^2^, Adjusted R^2^ and Normalized RMSE are computed for the training and testing data, and are given accordingly in Equations (15)–(17). The observations for the proposed approach are plotted in Figure 9. The proposed approach R^2^ was determined to be 0.9516 on training data and 0.9869 on testing data, which indicates that it obtained an accuracy of 95.16% on training data and 98.69% on testing data. On the training data and testing data, the adjusted R^2^ of the method proposed is 0.949 and 0.972, respectively. On train data, the model’s normalized RMSE score is 0.017, and for the testing data, it is 0.024.

**Figure 10:**
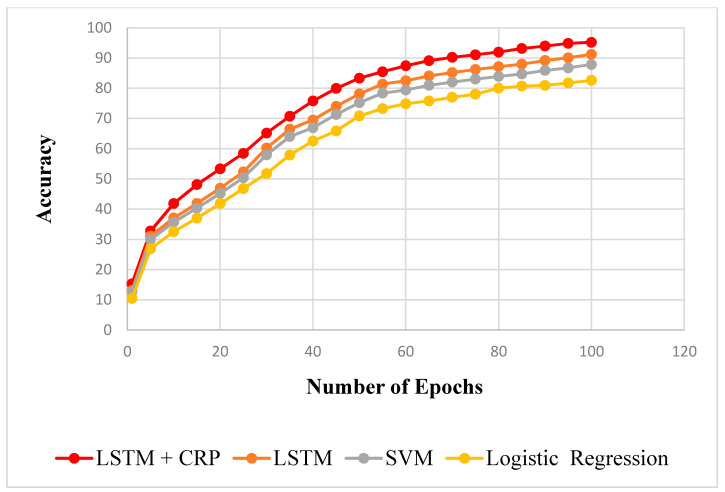
Train data R^2^ comparison.

**Figure 11:**
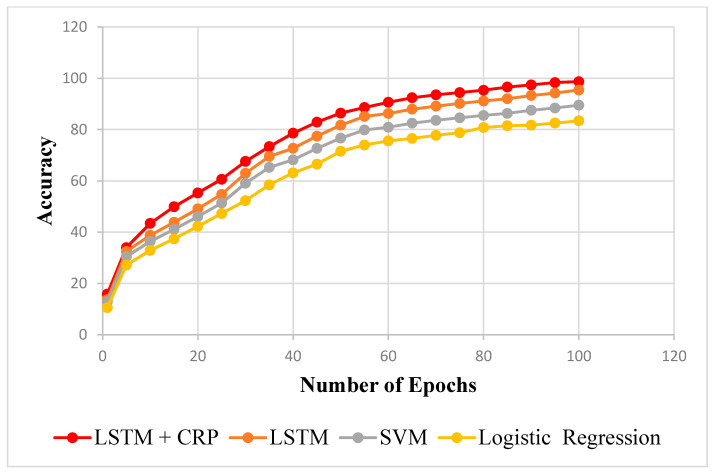
Test data R^2^ comparison.

In Table 5 and Table 6, the proposed framework is applied to compare the R^2^ scores of the training and testing data with the LSTM, SVM, and logistic regression. The LSTM model’s R^2^ scored 0.912 for training data and 0.954 for testing data. The SVM approach received scores of 0.878 for training data and 0.895 for testing data, respectively. The R^2^ value for the logistic regression was, respectively, 0.825 and 0.834 on the training and testing sets of data. However, the suggested strategy performs better compared to every other method. The values are plotted epoch-wise in Figure 10 and Figure 11 for training and testing data. According to this, it can be demonstrated that the proposed method, when related to other methods such as LSTM, SVM, and logistic regression, can predict insufficient resource failure with a higher degree of accuracy.

(d) Failure Prediction

The performance metric given in Equations (18) and (19) is for the applications of IoT of various sizes on varied fog devices (20–100) over a time frame of ninety hours, which includes meantime (to recover, before failure) (MTTR) and (MTBF), availability average (Ava avgr), failure prediction accuracy (fpa), and number of failures predicted (n_fp). The performance metrics with respect to MTTR, MTBF, Ava Avgr, fpa, and n fp for GCD workload for different sizes of fog nodes 20 to 100 over the timeframe of 200 min are reported in Table 7. The performance of metrics with regards to Precision (P), Recall (R), and F-measure are characterized by the prediction accuracy of several resources using the proposed technique to calculate the average prediction failure accuracy. The fog nodes’ resources and prediction errors directly but unevenly affect how well failures are predicted.

**Figure 12:**
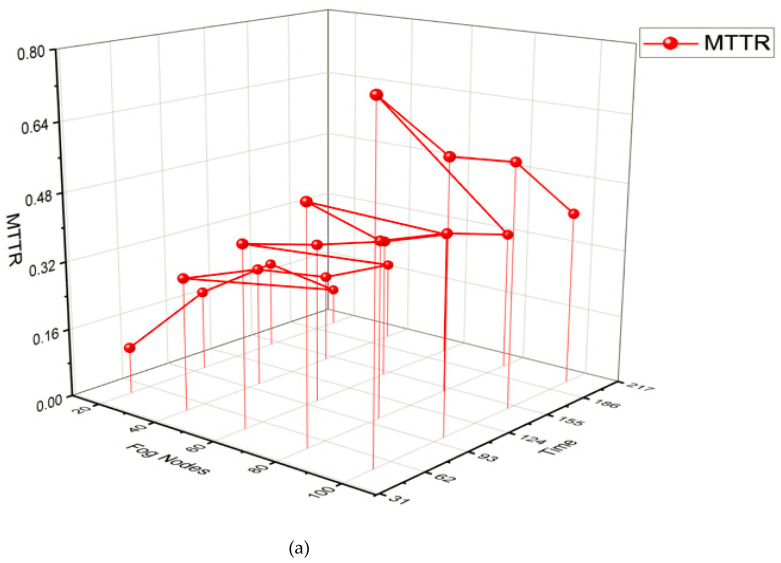

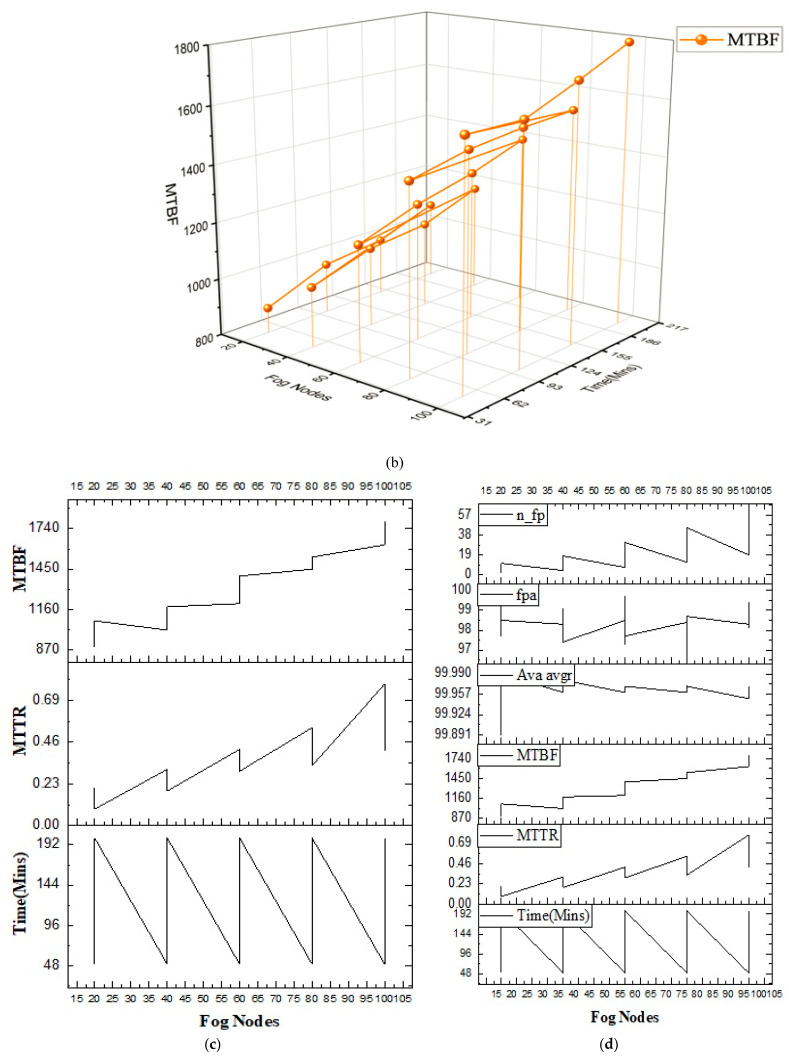
(**a**): Fog nodes, time, and MTTR. (**b**): Fog nodes, time, and MTBF. Represents the relationship between various parameters of MTBR and MTBF. (**c**): Fog nodes, time, MTTR, and MTBF. (**d**): Fog nodes, Time, MTTR, MTBF, Ava_Avgr, fpa, n_fp.

Additionally, the obtained MTBF and recovery rely on the number of failures. In fog node failure scenarios, MTTR is determined by the number of failures that are unpredicted, which differs from the number of fog nodes. The MTBF and recovery obtained throughout timeframe {k1, k2} and Equation (20) are used to calculate the corresponding availability values. Contrasting the evaluation of the proposed LSTM + CRP network’s failure prediction accuracy with that of the LSTM, SVM, and logistic regression. The MTBF and MTTR values that were obtained during the processing of IoT applications over the timeframe {k1, k2} influence availability. Table 7 displays the differences in the values of MTBF and MTTR that were noticed during the experimental simulation. As fog nodes and IoT applications are expanded, MTTR increases while MTBF decreases, indicating an inverse relationship between the two. Figure 12 shows the relationship between various parameters such as Fog Nodes, Time, MTTR, MTBF, Ava_Avgr, fpa, and n_fp.

(e) Significance Test Using Paired *t*-Test

To determine whether our suggested method is statistically significant, a paired *t*-test was run. Table 8 shows that the paired *t*-test has a *p*-value of 0.01, which is significantly lower than 0.05. It signifies that, at a 95% level of confidence, the enhancement of our proposed approach is statistically significant compared to that of LSTM.

## 6. Conclusions and Future Work

This work proposes a proactive prediction of failure of insufficient resources, using LSTM and CRP, which reduces the dependence on identifying and working on fog node resources. LSTM and CRP are used to enable effective failure predictions, allowing the system to predict faults. A specific resource that could fail while IoT applications are running is identified in the framework by providing knowledge about fog devices. The major steps considered to predict the proactive failure were to develop an LSTM model to determine the failure of fog devices, to further enhance the binary classification, CRP network policy is merged with LSTM to extract the resource that could fail. The experimental predictive model implemented using iFogSim indicates that there is a minimization of delay and processing time when compared to the standard LSTM, SVM, and logistic regression. With the addition of the CRP network policy, the accuracy of the model is improved, which brings in a promising approach to overcome failure by managing it before it occurs, so that IoT applications run smoothly. The experimental findings also demonstrate that MTTR and MTBF, which were determined from the processing of the failure prediction of the fog nodes across the time interval k1 and k2 vary with fog node availability. The variances in MTBR and MTBF values that were seen during the experimental simulation show that inverse correlations exist between MTTR and MTBF, with MTTR decreasing as MTBF increases. The future work in this research is a multidimensional perspective mechanism to tolerate faults based on the prediction of the faults in the devices.

## Figures and Tables

**Table 1:** Notations of Resources in Fog and IoT.

Fog Node Parameters	Description
FN_VM	Fog node virtual machine
FN_VMR	Fog node new virtual machine request for compute and storage resources
FNA_C+S	Fog node availability of computation and storage resources (CPU + RAM)
FN_UD	Fog node of uplink and downlink
FN_level	The level of the fog node
FN_pow	Fog node Power contains the busy power and idle power in watts
H_PR	The request which performs critical data processing and needs good response time
Y	IoT Applications
Y_C+S	Compute and storage resource requirements of each IoT application
R_CoFN	Fog node cost of a VM, when it is reallocated or rescheduled to another node
Schedule_Queue	VM requests are rescheduled back when their resources are available
FN_fault	The fog node does not cater to the needs of the IoT application and is not tolerable to faults and must be rescheduled from the queue
IoT_pow	IoT application requirement of power consumption

**Table 2:** Configuration of fog nodes.

System Device	Parameter	Value
Fog Nodes	Total fog nodes	10–25
Each Fog Node	Total VMs	2
	RAM (unit)	128–4000 (Mbps)
	CPU processing power (unit)	1000–2800 (MIPS)
	Power capacity (units)	1 (watt)
	Data storage capacity (unit)	1 (GB)
	The capacity of bandwidth (unit)	100–10,000 (Mbps)
	Total CPUs	1
	OS	Linux
	VMM–Hypervisor	Xen
Data Center	Data center	1
	Hosts	1

**Table 3:** Parameter settings of IoT application.

Parameter	IoT Applications Executed	Components of IoT Applications Length (MI)	File Size (MB)	Output Memory Size (MB)
Value	3–5	250–950	100–1550	15–55

**Table 4:** Fog node traces.

MIPS	RAM	Upload Bw	Download Bw	Level	Busy Power	Idle Power	Available CPU	Available Ram
44,800	16,000	100	10,000	0	1648	1332	2963.54	2073.37
2800	4000	10,000	10,000	1	106.339	63.67	2096.65	3074.78
2800	4000	10,000	10,000	1	97.339	84.54	623.48	1558.45
2800	4000	100	50	1	87.339	72.4333	2696.33	2135.91
1000	512	50	10	0	107.339	62.54	1581.53	3771.31
2000	750	8500	8500	1	97.349	82.5333	1723.21	1327.88
2000	1000	9000	8500	1	103.539	83.4333	965.55	2998.68
1500	3500	9500	9500	1	101.9	63.4333	2639.47	3801.73
2300	3300	7500	6000	1	91.339	83.4333	2695.63	1556.61
1750	3800	7600	7550	0	107.549	83.4333	797.44	3258.75
2100	2000	2000	1900	1	78.339	83.4333	677.98	3370.15

**Table 5:** Multiple epochs of training data R^2^ vs. various prediction techniques.

Epochs	LSTM + CRP	LSTM	SVM	Logistic Regression
10	41.8608	37.0656	35.6928	32.5523
35	70.7184	66.4632	64.0016	57.8932
50	83.322	78.0948	75.2024	70.8313
55	85.4496	81.3672	78.3536	73.2553
70	90.2016	85.1796	82.0248	76.9923
75	91.0548	86.1948	83.0024	78.0225
90	93.9384	89.1648	85.8624	80.9212
95	94.824	90.072	86.736	81.7292
100	95.1696	91.206	87.828	82.5978

**Table 6:** Multiple epochs of testing data R^2^ vs. various techniques.

Epochs	LSTM + CRP	LSTM	SVM	Logistic Regression
10	43.4112	38.7816	36.3792	32.8746
35	73.3376	69.5402	65.2324	58.4664
50	86.408	81.7103	76.6486	71.5326
55	88.6144	85.1342	79.8604	73.9806
70	93.5424	89.1231	83.6022	77.7546
75	94.4272	90.1853	84.5986	78.795
90	97.4176	93.2928	87.5136	81.7224
95	98.336	94.242	88.404	82.5384
100	98.6944	95.4285	89.517	83.4156

**Table 7:** Values of MTTR and MTBF.

Fog Nodes	Time (Mins)	MTTR	MTBF	Ava Avgr	fpa	n_fp
20	50	0.11	888.43	99.98	98.2	2
	100	0.19	976.12	99.98	97.7	5
	150	0.21	1001.89	99.89	99.2	8
	200	0.09	1078.45	99.99	98.5	11
40	50	0.31	1011.21	99.96	98.3	4
	100	0.28	1078.92	99.99	97.4	9
	150	0.21	1103.53	99.99	99.1	13
	200	0.19	1179.28	99.98	97.4	18
60	50	0.42	1202.16	99.96	98.5	7
	100	0.37	1276.29	99.97	99.7	16
	150	0.33	1329.03	99.97	97.3	22
	200	0.30	1399.45	99.97	97.7	31
80	50	0.54	1448.91	99.96	98.4	12
	100	0.41	1496.29	99.97	96.6	19
	150	0.38	1521.28	99.97	97.3	29
	200	0.33	1535.74	99.97	98.7	45
100	50	0.78	1623.12	99.95	98.3	19
	100	0.62	1622.88	99.96	98.8	26
	150	0.57	1705.28	99.96	99.4	49
	200	0.41	1794.48	99.97	98.1	62

**Table 8:** Paired *t*-test for significant test.

	Accuracy of the Proposed Approach	Accuracy of the LSTM Approach
Mean	0.98	0.84
Observations	5	5
Variance	0.0001	0.0009
Mean Difference Hypothesized	0	-
df	2	-
Pearson Correlation	−0.16423367	-
t Stat	4.6690876545	-
One tail when P(T ≤ t)	0.08679823	-
Two tail when P(T ≤ t)	0.17895624	-
T critical two tail	3.3198679	-

## Data Availability

Not applicable.
